# Activation of hippocampal MEK1 contributes to the cumulative antinociceptive effect of electroacupuncture in neuropathic pain rats

**DOI:** 10.1186/s12906-016-1508-z

**Published:** 2016-12-15

**Authors:** Yong-Hui Gao, Cheng-Wen Li, Jun-Ying Wang, Yu Kan, Lian-Hong Tan, Xiang-Hong Jing, Jun-Ling Liu

**Affiliations:** 1 Department of Physiology, Institute of Acupuncture and Moxibustion, China Academy of Chinese Medical Sciences, Beijing, 100700 China; 2 College of Acupuncture & Moxibustion and Tui-na, Hunan University of Chinese Medicine, Changsha, 410208 Hunan Province China; 3 The First Affiliated Hospital of Hunan Traditional Chinese Medical College, Zhuzhou, 412012 Hunan Province China; 4 Meridian Research Center, Institute of Acupuncture and Moxibustion, China Academy of Chinese Medical Sciences, Beijing, China

**Keywords:** Acupuncture analgesia, Neuropathic pain, Cumulative effect, Hippocampus, Mitogen-activated protein kinase kinase 1

## Abstract

**Background:**

Electroacupuncture (EA) intervention can relieve a variety of pain; however, optimal EA protocols have not been clearly determined. In addition, although central mitogen-activated protein kinase kinase (MEK) signaling has been shown to be involved in the antinociceptive effect of acupuncture stimulation, its characteristics at different time-points of EA intervention have not been fully elucidated. Therefore, the present study investigated the relationship between the effects of different numbers of EA intervention sessions and the activation of MEK1 in the hippocampus and hypothalamus in a rat model of neuropathic pain.

**Methods:**

After ligation of the left sciatic nerve, which induces chronic constriction injury (CCI), the acupoints Zusanli (ST36) and Yanglingquan (GB34) were applied. The thermal withdrawal latency of the hind paw was used to evaluate the effect of EA on pain thresholds. Intra-hippocampus microinjection of PD98059, a MEK inhibitor, was performed to validate the involvement of MEK in EA analgesia. The hippocampus and hypothalamus were harvested to examine the phosphorylation levels of MEK (pMEK) by western blotting.

**Results:**

In CCI rats, the thermal pain threshold of the affected hind paw decreased significantly relative to the control. Following subsequent daily EA interventions, CCI-induced ipsilateral hyperalgesia was markedly improved from day 4 and the analgesic effect of EA lasted 3 days after cessation of EA. Four sessions of EA markedly suppressed CCI-induced decrease of hippocampal pMEK1 (normalized to the total MEK level). In contrast, successive sessions of EA intervention gradually down-regulated the CCI-induced up-regulation of hypothalamic pMEK1 along with the increase numbers of EA intervention. However, EA did not exert the same analgesic effect after microinjection of PD98059 into the contralateral hippocampus during the first 3 days of EA intervention.

**Conclusions:**

EA intervention can induce time-dependent cumulative analgesia in neuropathic pain rats after 4 successive sessions of daily EA intervention, which is at least in part related to the activation of hippocampal MEK1.

## Background

Neuropathic pain is a refractory chronic pain syndrome with a complicated mechanism that currently has no effective therapy. Acupuncture therapy, a method of traditional Chinese medicine, has received worldwide attention as a strategy to relieve pain [1–3]. Clinical practice demonstrates that multiple sessions of acupuncture treatment (not a single session) may induce an obvious analgesic effect [4–6]. However, the number of acupuncture treatment sessions required for effective pain relief is unclear.

Several lines of evidence demonstrated that the short-term effect of a single session of acupuncture stimulation differs from the cumulative effect of multiple sessions of acupuncture stimulation, which may depend on the differential modulation of immunologic factors and neurotrophic signaling molecules [7–9]. Our previous study showed that the cumulative analgesic effect of repeated electroacupuncture (EA) intervention in neuropathic pain rats is closely associated with its regulatory effects on natural killer cells as well as some splenic and plasma cytokines [10]. Some findings suggested that multiple sessions of EA induced up-regulation of angiotensin I and II and the *δ*-opioid receptor in cerebral ischemia rats [11, 12]. However, the central regulation mechanism of the cumulative analgesic effect of multiple sessions of EA intervention remains unknown, particularly under chronic pain conditions. Answering these questions will provide new evidence and shed new light on the targets of acupuncture intervention.

There was evidence that acupuncture stimulation of remote acupoints induced responses in various brain regions to exert its function [13], and many nuclei of the limbic system and brainstem have been experimentally confirmed to mediate acupuncture information [14]. Our previous study demonstrated that in chronic constriction injury (CCI)-induced neuropathic pain rats, the cumulative analgesic effect of repeated EA stimulation of Zusanli (ST36)-Yanglingquan (GB34) is closely associated with its effects in upregulating the decreased hippocampal synaptophysin immunoreactivity and improving synaptic plasticity in the hippocampus [15]. Blocking neural transmission along the major afferent or efferent hippocampal pathways reduced pain behaviors [16, 17]. These findings may support the notion that hippocampal formation plays an important role in both pain and EA perception. Our initial work showed that the hippocampus participated in the cumulative analgesic effect of multiple sessions of EA intervention, and many molecules in this brain region were implicated in this process [18]. Among them, mitogen-activated protein kinase kinase 1 (MEK1) signaling has warranted special attention.

MEK1 is crucial in hippocampal-dependent learning, memory, and synaptic plasticity [19]. A close relationship has been documented between neuropathic pain and activation of MEK1. For example, MEK1 in dorsal horn neurons of the spinal cord and in the amygdala contributed to pain [20]. In our previous study, proteins differentially expressed in the hippocampus and hypothalamus were identified using two-dimensional gel electrophoresis and matrix-assisted laser desorption/ionization time-of-flight mass spectrometry. We found that after CCI, MEK1 was down-regulated in the hippocampus and up-regulated in the hypothalamus. However, following 12 sessions of EA intervention, MEK1 in both regions recovered to normal levels [18, 21]. We extended that work in the present study to clarify the differential effects of multiple and single EA intervention sessions in CCI rats. We examined the changes of MEK1 activation in the hippocampus and hypothalamus after different EA intervention sessions. In addition, we assessed the involvement of MEK1 in the cumulative analgesic effect of multiple sessions of EA intervention by intra-hippocampal injection of a MEK1 inhibitor in neuropathic pain rats.

## Methods

### Animals

Male Wistar rats (240–300 g) were obtained from the Experimental Animal Center of the Peking Union Medical College (Beijing, China) and housed within the animal care facilities in the Institute of Acupuncture and Moxibustion, China Academy of Chinese Medical Sciences. Food and water were available ad libitum. The animals were allowed to adapt to the experimental conditions in the laboratory for at least 2 h before pain testing. All experimental procedures were approved by the Institute of Acupuncture and Moxibustion of China Academy of Chinese Medical Sciences and were identical to those recommended in the *Guidelines for Laboratory Animal Care and Use* from the Chinese Ministry of Science and Technology (2006). Efforts were made to minimize the number and suffering of the animals used.

### Experimental design

The study consisted of the following two experiments: Experiment 1 was designed to examine the effects of EA on MEK1 activation. For this experiment, rats with CCI were randomized into eight groups (*n* = 10 in each group): sham operation control (CON), CCI model, and CCI plus an increasing number of daily EA treatment (EA2d, EA4d, EA6d, EA8d, EA10d, and EA12d); Experiment 2 was designed to validate the effects of MEK1 depletion on the cumulative effects of EA intervention. Rats that received CCI and implantation of guide cannulas in the contralateral hippocampus were divided into four groups and treated with either a microinjection of dimethyl sulfoxide (DMSO for control; two groups, CCI + DMSO, CCI + DMSO + EA) or PD98059 (a MEK1 inhibitor, two groups, CCI + PD98059, CCI + PD98059 + EA). To observe the different effects of PD98059 at different stages of EA intervention, a 3-day cycle hippocampal microinjection was given on the first 3 days of EA or given on day 8, 9, and 10 of EA separately (*n* = 10 in each group). To examine the effect of a combination of microinjection and EA, rats received a microinjection 4.5 h prior to EA stimulation. To clarify the effect of PD98059, a CON + PD98059 group was added (*n* = 10). Randomization scheme was created using the standard  =  RAND() function in Microsoft Excel.

### CCI pain model and behavioral tests

The CCI model was established by unilaterally ligating the sciatic nerve, as previously reported [22]. Briefly, under anesthesia (25% urethane plus 1.5% chloralose, 0.4 mL/100 g body weight) and using routine sterile procedures, the left sciatic nerve was exposed at the mid-thigh level by blunt dissection through the biceps femoris. Four constrictive ligatures (4–0 non-absorbable suture) equally spaced by approximately 1 mm were tied around the nerve at the distal end close to the bifurcation site. The ligatures were tightened until a moderate muscular contraction of the leg was observed. For the CON group, the rats underwent the same procedure but without nerve ligation.

One day before ligation, 10 days after ligation, and everyday within 0.5 h before EA stimulation, the paw withdrawal latencies (PWL, i.e., thermal pain threshold) of both hind paws were determined using a 37370 Algesia Detector (Ugo, Italy). A radiant heat source was focused on the plantar surface of a hind paw, and a light intensity was preset to obtain a baseline latency of approximately 25 s. Each rat underwent three trials with a 5-min interval, and the mean value of three trials was used as the PWL.

### Electroacupuncture

According to traditional Chinese medicine theory, Zusanli (ST36) and Yanglingquan (GB34) are considered the most effective acupoints for treating low back pain and are commonly used to study the acupuncture effects on various physiological regulatory and control systems in modern scientific research. ST36 is located 5 mm beneath the capitulum fibulae and lateral posterior to the knee joint, and GB34 is approximately 5 mm superior-lateral to ST36 [18]. In the present study, the animals in the EA group were treated with bilateral ST36 and GB34. The acupoints were punctured with stainless steel filiform needles (diameter 0.35 mm, length 40 mm, Huatuo; Suzhou Medical Appliance Manufactory, Jiangsu, China) to a depth of approximately 2–3 mm, and stimulated electrically for 30 min using a Han’s EA Stimulator (LH202; Neuroscience Research Center, Peking University, Beijing, China). The EA stimulation parameters were 1 mA and 2 and 15 Hz alternating frequencies (automatically shifting between 2 Hz and 15 Hz stimulation for 3 s each), respectively. EA stimulation started from the 10^th^ day after CCI. During EA stimulation, the animals were awake and constrained with a special cloth bag. As neither the ipsilateral nor the contralateral PWLs were influenced by the repeated constraining process in CCI rats, the non-EA groups (CCI and CON groups) underwent the same constraining procedure, but without EA stimulation.

### Microinjection procedure

Under anesthesia with urethane and chloralose, after CCI operation, two 6-mm-long stainless steel guide cannulas (Small Parts Inc., Hialeah, Florida, USA) were stereotaxically implanted into the contralateral hippocampus (3.3–3.6 mm posterior to the bregma, 2.4–2.7 mm lateral to the midline, and 3.0–3.5 mm below the cortical surface) based on the coordinates in the atlas completed by Paxinos and Watson [23], with the tip retained about 2 mm above the dura. The guide cannulas were anchored to the cranium with dental cement. The animals were allowed to recover for 10 days. The guide cannulas were plugged with a stainless steel stylet, which was removed before drug administration. PD98059 (Abcam, New Territories, HK) dissolved in 5% (v/v) DMSO (Abcam) was administered to the PD98059 groups. The DMSO groups received the same volume of 5% (v/v) DMSO as control. Drugs were administered into the hippocampus through the guide cannulas using an injection needle (gauge 27) connected by polyethylene tubing to a 10-μL Hamilton microsyringe while the rats were anesthetized under isoflurane anesthesia. PD98059 (10 μg/10 μL) or DMSO (5% (v/v)/10 μL) was injected over 3 min, 4 h prior to behavioral testing.

**Fig. 1:**
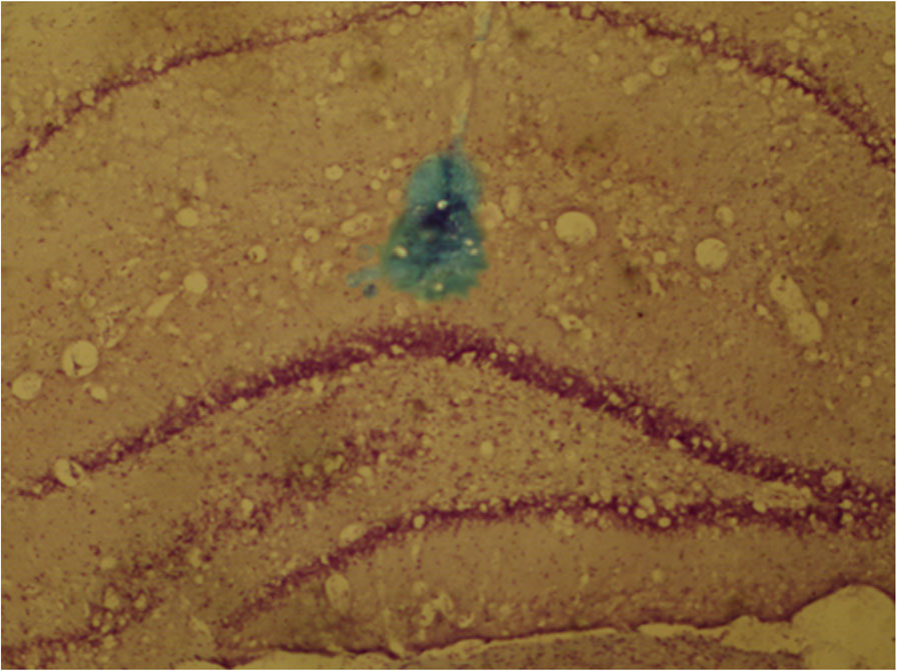
Representative photomicrograph of the location of the injection site in the rat hippocampus

At the end of each set of experiments, the microinjection sites of one rat in each group were marked with 2 μL of a saturated solution of Pontamine sky blue (Sigma Chemical Co. St. Louis, MO, USA) to determine the distribution of the injection. After fixation with 10% (v/v) formalin, the brain was sectioned and counterstained with cresyl violet. The microinjection sites were histologically verified and plotted based on the Paxinos and Watson stereotaxic atlas coordinates. A representative microinjection site is shown in Fig. 1. For all animals examined, the cannula tips were located just in the target region, and the injection tracks were limited to the CA region or dentate gyrus of the hippocampus.

### Tissue harvest and western blot analysis

After termination of the behavioral test, rats in each group were deeply anesthetized and killed by decapitation. The right and left hypothalamus and hippocampi were removed rapidly on an ice plate and then washed with normal saline. Tissue extracts were obtained using mechanical tissue disruption in RIPA lysis buffer with 1× Complete Anti-Protease Cocktail (Roche, Indianapolis, IN, USA), 1 mM PMSF, and incubation for 10 min at 4 °C. Lysates were cleared by centrifugation at 10,000 g for 10 min at 4 °C. Total protein (30 μg) was loaded onto 10% (w/v) gels for sodium dodecyl sulfate polyacrylamide gel electrophoresis and transferred onto polyvinylidene membranes (Millipore, Billerica, MA, USA) for western blot analysis. Membranes were then incubated with primary antibodies (anti-MEK1, 1:1000; anti-phospho-MEK1, 1:800; anti-GAPDH, 1:500; Cell Signaling, Beverly, MA, USA) overnight at 4 °C with 2% (w/v) bovine serum albumin in PBST. The membranes were then washed three times with PBST (10 min each time), incubated with the appropriate horseradish peroxidase-conjugated secondary antibody for 2 h at room temperature, followed by three washes with PBST. Immunoreactive bands were then revealed by enhanced chemiluminescence (Amersham Biosciences UK Limited, Buckinghamshire, England) using standard x-ray film (Eastern Kodak Co., Rochester, NY, USA). The relative intensities of the detected protein bands were analyzed with a Personal Densitometer SI (Amersham Biosciences) linked to ImageQuant 5.2 software (Amersham Biosciences).

### Statistical analysis

SPSS 17.0 software was used to perform the statistical analysis. The results are expressed as the mean ± SD. PWLs were analyzed using two-way repeated measures analysis of variance (ANOVA) with one between-subject factor (EA intervention) and one within-subject factor (time) followed by the least significant difference (LSD) analysis. Western blot data were analyzed using one-way analysis of variance and the LSD test was performed to compare the differences between two groups. All *P* values were derived from two-sided tests, and *P* < 0.05 was considered statistically significant.

## Results

### Effects of EA treatment on pain threshold

**Fig. 2:**
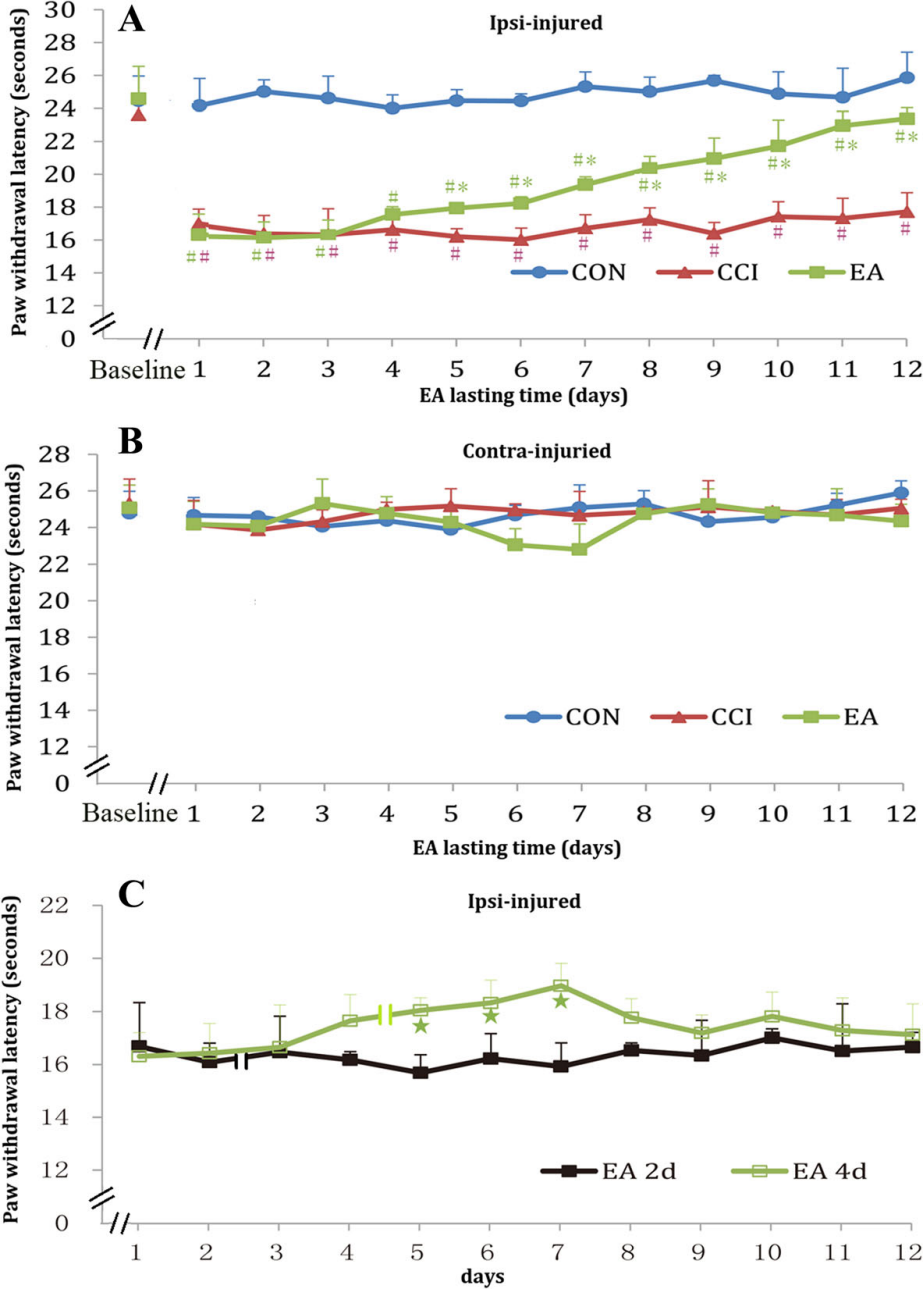
Effect of EA stimulation on PWL in CCI rats. **a** ipsilateral and **b** contralateral paw. **c** The lasting effect of the number of EA interventions (2 or 4 days of treatment) on PWL. The double-slash indicates EA intervention was discontinued. Data are presented as the mean ± SD; *n* = 10 in each group. ^#^ *P* < 0.05 vs. CON group, *P < 0.05 vs. CCI group, ^☆^ *P* < 0.05 vs. EA2d group

Baseline measures of PWL to radiant heat stimulation on both hind paws did not differ among the CON, EA, and CCI groups. Ten days after surgery, strong thermal hyperalgesia developed in the ipsilateral hind paw. Two-way repeated measures ANOVA showed no significant interaction between EA intervention and time-points (*P* = 0.083) and a significant difference among the CON, EA and CCI groups (*P* < 0.01). Following four successive daily EA interventions, CCI-induced ipsilateral hyperalgesia was markedly suppressed and the PWL displayed a time-dependent increase from the 4^th^ day onwards (Fig. 2a). Furthermore, after four successive sessions of EA intervention, the analgesic effect of EA lasted at least 3 days after cessation of EA. However, after 2 successive sessions of EA intervention, the lasting effect was not observed (Fig. 2c). PWL in the contralateral hind paw was not different among the three groups and remained stable during the intervention process (Fig. 2b).

### Effect of EA treatment sessions on pMEK1 protein

**Fig. 3:**
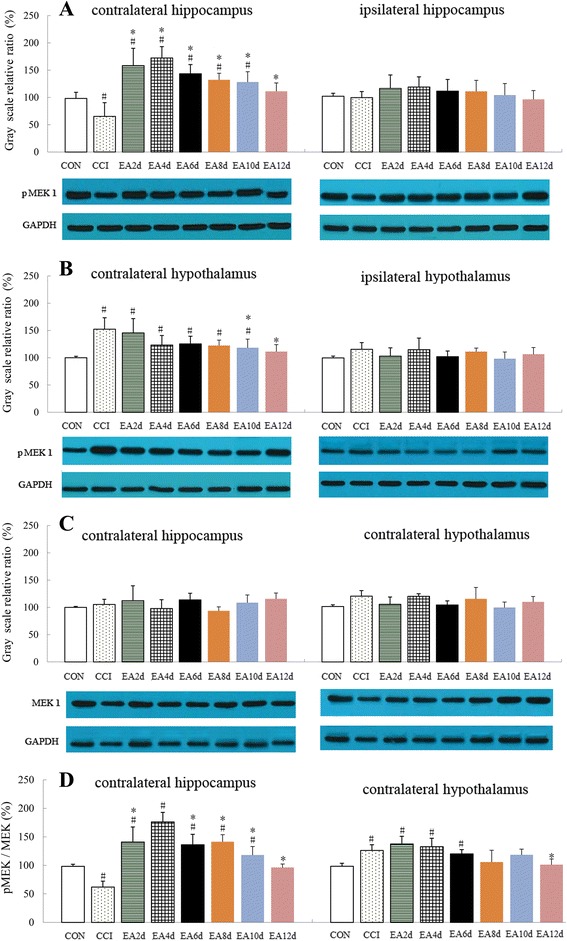
Effect of EA stimulation on pMEK1 and MEK1 expression in CCI rats. **a** The level of pMEK1 in the hippocampus **b** The level of pMEK1 in the hypothalamus **c** The expression of MEK1 in the hippocampus and hypothalamus contralateral to the injured sciatic nerve **d** The fold change for the gray scale ratio of pMEK normalized to the total MEK. Data are presented as the mean ± SD; *n* = 10 in each group. ^#^ *P* < 0.05 vs. CON group, *P < 0.05 vs. CCI group

To examine whether MEK1 is activated in the hippocampus and hypothalamus after CCI and EA, we performed a western blot analysis with antibodies targeting the total MEK1 expression and the dually phosphorylated MEK1 (pMEK1, Ser217/221) because phosphorylation at Ser-217 and Ser-221 positively regulates MEK1 activity. In hippocampal tissue contralateral to the CCI limb, we observed a reduction of pMEK1 in rats with CCI compared with the sham operation control group. This reduction was significantly reversed from day 2 to 4 of EA treatment, and MEK1 activation in rats with EA was higher than that in the CON group animals (*P* < 0.01). After 6 days' EA stimulation, the contralateral hippocampal pMEK1 was significantly decreased and nearly returned to the control level after 12 sessions of EA (Fig. 3a). After normalizing pMEK1 levels to total MEK1 levels (pMEK1 versus MEK1), the same results were obtained as above (Fig. 3d). No significant changes in pMEK1 expression were noted in the ipsilateral hippocampus throughout the time course of EA (Fig. 3a) and no significant changes were found in total MEK1 expression levels (Fig. 3c).

Contralateral hypothalamic pMEK1 protein expression was considerably increased following CCI and gradually decreased with an increase of EA intervention sessions and finally returned to the control level after 12 EA sessions (Fig. 3b and d). Similar results to the ipsilateral hippocampus were seen, and no changes in pMEK1 expression were found in the hypothalamus ipsilateral to the affected limb following CCI and EA intervention (Fig. 3b).

### Effect of EA combined with PD98059 on pain threshold

**Fig. 4:**
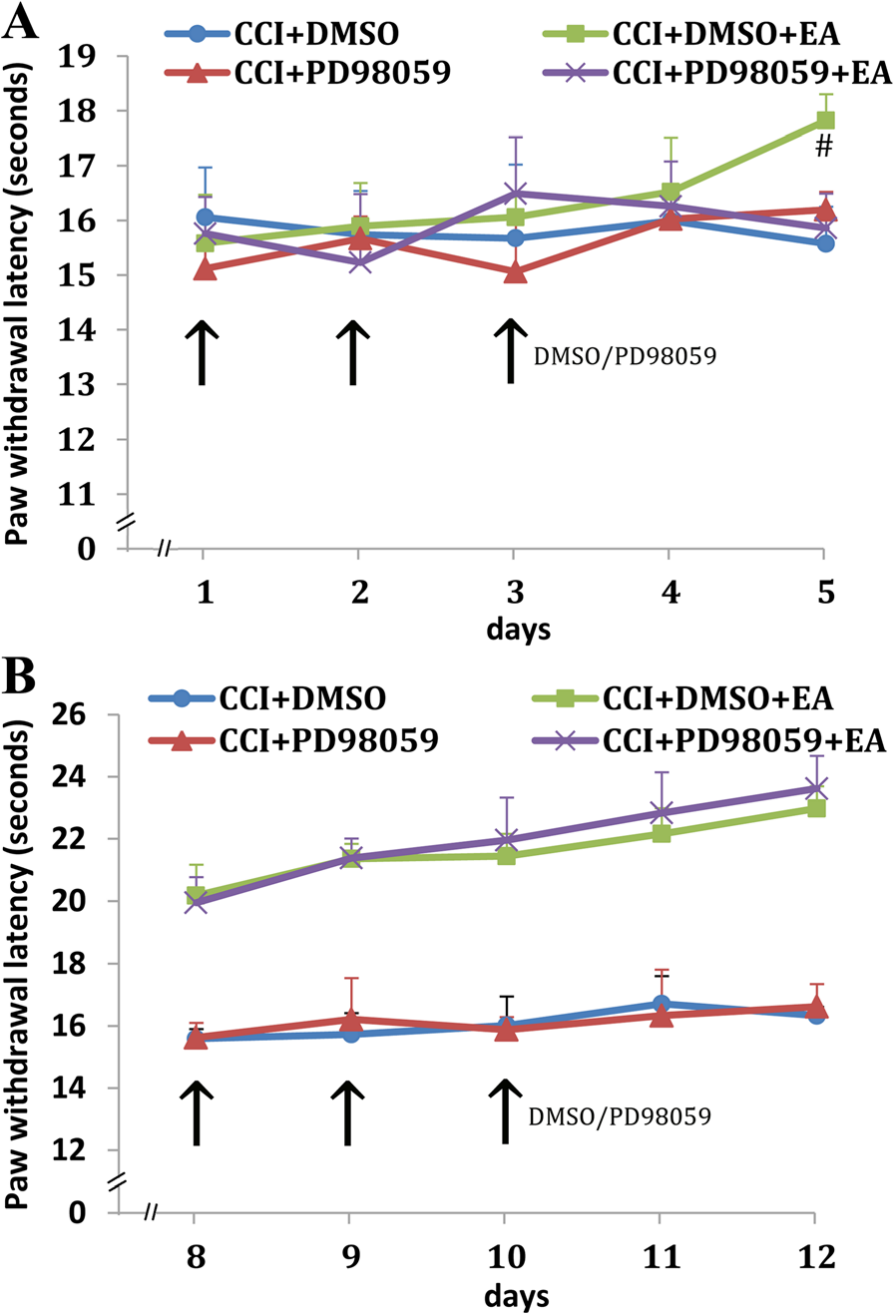
Effect of intra-hippocampal microinjection of the selective MEK1 inhibitor PD98059 at different time-points on PWL in a rat model of neuropathic pain. **a** Microinjection was given on the first 3 days of EA, **b** microinjection was given from the 8th day of EA. The *black arrowheads* indicate the day of PD98059 or DMSO administration. Data are presented as the mean ± SD; *n* = 10 in each group. ^#^ *P* < 0.05 vs. CCI+ PD98059 + EA group

To detect the different role of MEK1 at different stages of EA intervention, the selective MEK1 inhibitor PD98059 was injected into the contralateral hippocampus on the first 3 days or on day 8, 9, and 10 of EA treatment separately. For rats in the CCI group, after PD98059 administration on day 1, 2, and 3 or on day 8, 9, and 10, the thermal pain thresholds of the affected hind paw showed no apparent changes compared with those following administration of DMSO (Fig. 4a, b). After PD98059 administration in rats from the EA intervention group on day 1, 2, and 3, the thermal pain thresholds of the affected hind paw showed no apparent changes compared with those of administration of DMSO. However, after completion of the first 3-days’ administration, the PWL was increased continually in the DMSO group while the PWL in the PD98059 group showed no apparent increase during the following EA days. On the 5^th^ day of EA intervention, the PWL in the DMSO group was significantly higher than that in the PD98059 group (*P* = 0.036, Fig. 4a). After administration of PD98059 on day 8, 9, and 10 of EA interventions, the PWLs were slightly increased compared with those in rats of the DMSO group. However, no significant difference was observed (Fig. 4b).

### Effect of EA combined with PD98059 on the pMEK1 protein

To determine the effect of PD98059 microinjections on EA functions in regulating MEK expression, we examined the levels of pMEK1 in the contralateral hippocampus and hypothalamus after completion of each cycle of the 3-day PD98059 injection, namely on the 3^rd^ and 10^th^ day of EA intervention, as well as after 2 days’ rest (i.e., on the 5^th^ and 12^th^ day of EA treatment).

**Fig. 5:**
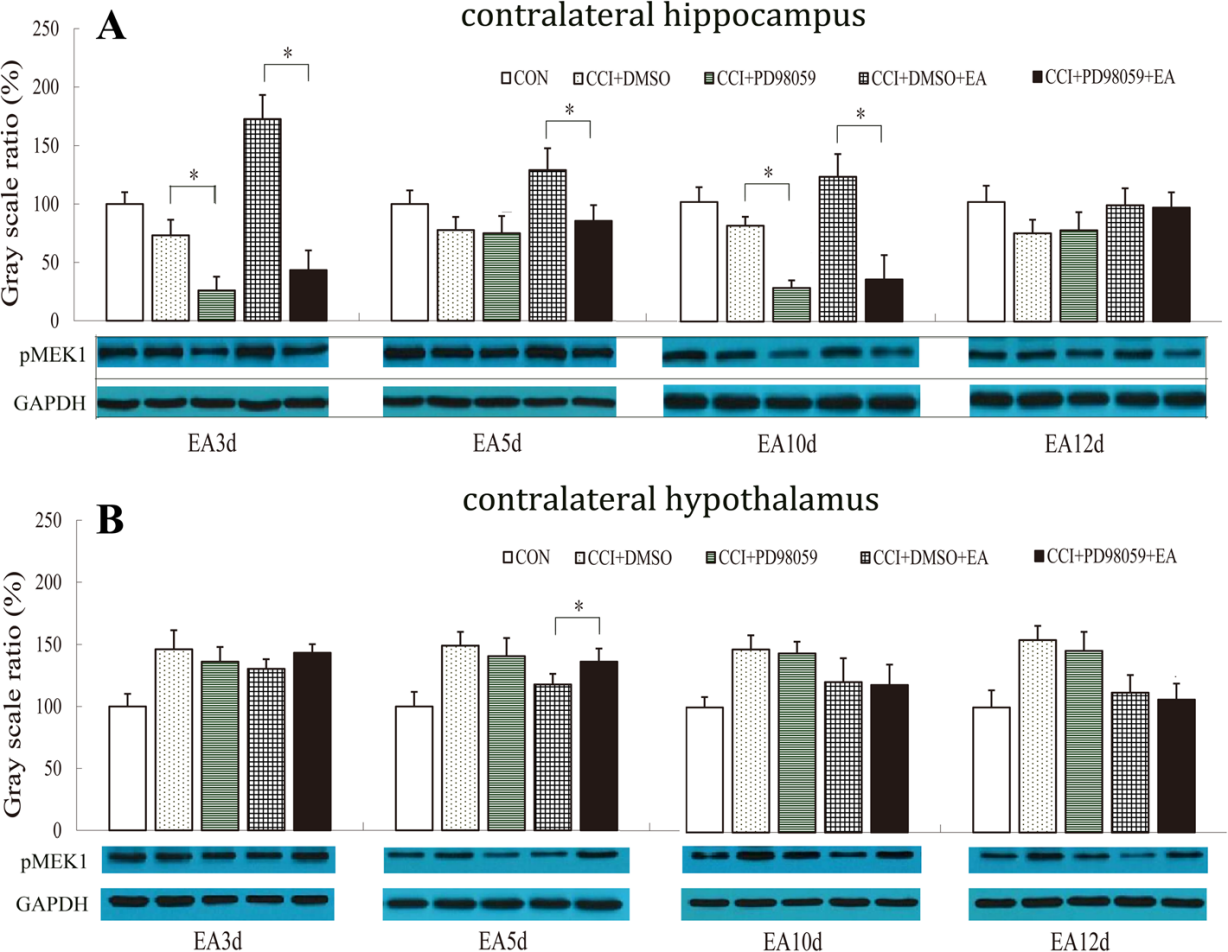
Effect of the MEK1 inhibitor PD98059 on **a** hippocampal and **b** hypothalamic pMEK1 in a rat model of neuropathic pain. Data are presented as the mean ± SD; *n* = 10 in each group. * *P* < 0.05

After completion of PD98059 injection, on day 3 and 10 of EA intervention, the pMEK1 in the contralateral hippocampus were considerably down-regulated in the CCI+ PD98059 and CCI + EA + PD98059 groups in comparison with those of the corresponding DMSO control groups (*P* < 0.05, Fig. 5a). After 2 days’ rest, on day 12, the decreased levels of hipppocampal pMEK1 protein in the two PD98059 administration groups (i.e., CCI+ PD98059 and CCI + EA + PD98059) were comparable tothose of  their corresponding DMSO control groups (*P* > 0.05). However, on day 5, the pMEK1 in the CCI + EA + PD98059 group in the contralateral hippocampus remained lower than that in the EA + DMSO group.

After hippocampal microinjection of MEK1 antagonist PD98059, there were no apparent differences in the levels of hypothalamic pMEK1 protein between the PD98059 groups and their corresponding DMSO control groups on days 3, 10, and 12 (*P* > 0.05,). However, after hippocampal microinjection of PD98059, the decreased level of pMEK1 in the contralateral hypothalamus induced by 5 days'  EA treatment was no longer observed, being significantly higher than that in the EA + DMSO group (*P* = 0.041, Fig. 5b).

## Discussion

Although it has been reported that EA intervention effectively alleviated pain, the optimal EA treatment protocol remains unclear. In this study, no significant changes were observed in the PWL during the first three sessions of EA intervention, meaning no remarkable pain relief at that time, but a marked increase of the pain threshold was observed after four sessions of EA intervention, presenting a time-dependent pain relief thereafter. This was consistent with our previous study [10]. We also determined the duration of the post-effect of EA treatment. Our results indicated that after four successive sessions of EA, even when the EA treatment was discontinued, the increased pain threshold could last for 3 days. However, after only two sessions of EA intervention, no marked post-effect was found. Hence, these results suggest an apparent cumulative antinociceptive effect of four successive sessions of EA treatment in chronic neuropathic pain rats.

Pain is characterized as a multidimensional experience, involving nociception, emotional, and cognitive aspects. The hippocampus, a major component of the limbic system, has been shown to contribute to pain-related memory and emotional disorders [24, 25]. Recent findings suggested an association between chronic pain and alterations in the hippocampus [26, 27]. However, the potential mediators associated with these alterations have not been determined. Several lines of evidence demonstrated that noxious stimulation induced an alteration in hippocampal intracellular signaling cascades, and resulted in synaptic transmission modifications affecting neuronal excitation in the hippocampus [27–29]. These modifications lead to amplification of pain signaling, that is thought to be critical for the recovery and survival of an organism following injury. Among them, MEK1 signaling has been shown to be implicated in hippocampus-dependent memory formation. Impaired memory, a prominent complaint of patients with chronic pain, substantially contributes to pain-related disability [30, 31]. Based on our initial results that hippocampal MEK1 was altered differently after CCI and EA intervention, here, we tested whether hippocampal MEK1 was involved in the cumulative antinociceptive effect of EA intervention. We found that the number of EA treatment sessions, fewer than four sessions versus more than four sessions, was closely associated with different patterns of pMEK1 in the hippocampus. In the first four EA intervention sessions, hippocampal pERK1 was significantly increased in comparison to the CCI and control animals. After the cumulative analgesic effect of EA was present, hippocampal pMEK1 levels decreased gradually. These data suggest that MEK1 is activated in the hippocampus contralateral to the nerve injury side following four times of EA intervention, and this increase in MEK1 activity may initiate a cumulative effect of EA treatment. Previously reported gene screening results suggest that the discrepancy between the effects of multiple and single EA treatments on brain ischemia/reperfusion are possibly caused by their differential modulation on gene expression, such as brain-derived neurotrophic factor and signal transducer-related genes [4]. This proposal is consistent with our data. In addition, after suppressing MEK1 with its specific antagonist in the early stage of the successive EA intervention, the time-dependent decrease of PWL following four successive sessions of EA did not appear as expected, indicating that MEK1 may contribute to the initiation of the cumulative acupuncture analgesia. After the completion of each cycle of the 3-day PD98059 injections, namely on the 3rd and 10th day of EA intervention, the expression levels of pMEK1 in the contralateral hippocampus were considerably down-regulated. In the first four EA intervention sessions, no obvious lasting effect of EA was shown, so the effect of pMEK1 decreasing with PWL was not obvious during this period. But with a marked increase in the pain threshold after four sessions of EA intervention, the effect of pMEK1 decreasing with EA was obvious, appearing on day 5. However, we do not interpret our results to mean that PD98059 needs such a long time to play a role. Rather, the first 3-day PD98059 injections disrupted the cumulative EA treatment effect after four EA intervention sessions. These results indicate the different role of MEK1 at different EA intervention sessions. That is, suppression of MEK1 at the early stage of EA intervention may decrease the EA effect, whereas after multiple EA sessions, the effect of EA interventions may become pMEK1-independent.

Some studies have demonstrated that EA intervention can modulate the function of interneurons in the hippocampus and enhance long-term potentiation in the rat [32]. At the neural level, pain is expected to lead to stronger modulations in memory-related brain regions of the medial temporal lobe, such as the hippocampus, during encoding [33, 34]. These findings may give rise to the idea that a “pain memory” is encoded within the nervous system [35]. In daily life activities, humans and animals are constantly exposed to simultaneous inputs from different sensory modalities. In this complex environment, different stimuli commonly compete for limited processing resources [36]. Under such conditions, MEK1 signaling shows differential expression patterns based on the nociceptive stimulus. A study by Seo and colleagues showed that in acute or transient pain, the kinases downstream of MEK1 were increased, and single-stimulus pain upregulated hippocampal MEK signaling, which is critical for long-term information storage as well as neuronal adaptive responses [37]. However, with regard to chronic pain, there is a marked decrease in phosphorylation and gene expression of the components of MEK1 signaling [26]. These results demonstrate that the hippocampus processes pain-related information differently based on the pain modality. The animal is thought to undergo some important psychophysical aspects of pain, such as an enhanced response to a normally noxious stimulus and an allodynia response to a normally innocuous stimulus during CCI [38]. A single or few sessions of acupuncture intervention may also be regarded as acute nociceptive stimuli under CCI conditions and would compete with CCI-induced pain processing, such as MEK1 signaling, to inhibit chronic pain inducing. This may explain why in the first four sessions of EA intervention, MEK1 activation in rats with EA was higher than that in CCI animals. A study showed that the cumulative effects in the process of repeated acupuncture stimulation may be an interesting characteristic of bimodal habituation [9]. In other words, information induced by repeated EA stimulation may form a type of habitual memory in the hippocampus. Thus, it is feasible to think that the acupuncture memory may compete with the pain memory. Our study showed that a single EA stimulus induced upregulation of hippocampal pMEK1, which may be a major molecular mechanism for converting immediate EA-induced signaling to repeated treatment-induced EA signaling. After several sessions of EA treatment, with the neuronal adaptive response and the formation of habitual memory, the acute-specific effects of EA intervention may be gradually decreased, progressively decreasing the activation of MEK1 to normal levels. PWL is gradually increased after repeated EA treatment and returns to the normal level after 12 sessions of EA treatment. In parallel, pMEK is gradually decreased to the control level after 12 sessions of EA treatment. This suggests that activation of hippocampal MEK1 is implicated in the effect of EA. On day 12, with the increased number of EA sessions, the PWL and MEK1 levels return to normal values. Because MEK1 signaling is necessary for cell survival, the reduction of MEK1 signaling observed in the hippocampus of chronic neuropathic pain animals may influence the reduction of neurogenic cells and decrease the hippocampal volume [26]. These unique hippocampal disruptions are specific cellular correlation of the observed cognitive and emotional problems seen both in animal models of chronic pain and human patients with pain. Therefore, targeting the reversal of these systematic changes in chronic pain may provide an opportunity to permanently alleviate chronic pain. This is further supported by the result of the present study that the changed MEK1 activation induced by repeated EA intervention contributed to the increase of pain threshold in CCI rats. Therefore, further experiments should be performed to explore the exact role of MEK1 activation and how MEK1 interacts with other molecules following different sessions of EA.

After different EA treatment sessions, the alteration in pMEK1 level in the hippocampus was different from that in the hypothalamus. The pMEK1 in the hippocampus contralateral to the nerve injury was markedly increased after few EA treatment sessions, but decreased after multiple EA sessions. In contrast, compared with the CCI animals, hypothalamic pMEK1 level was decreased after a single EA treatment and decreased further with multiple EA sessions. These results indicate that the MEK pathways in the hippocampus and hypothalamus might contribute to the effects of EA treatments via different transmission mechanisms. After suppression of hippocampal MEK1 with PD98059, the decrease of hypothalamic pMEK1 on days 3 and 5 of EA treatments was no longer apparent, indicating that hippocampal MEK1 may influence the phosphorylation levels of hypothalamic MEK1. Although the mechanism mediating the effect of hippocampal MEK1 on hypothalamic MEK1 was not determined in our study, a more thorough understanding of the mechanisms underlying these phenomena will contribute to the refinement of therapeutic strategies for a variety of pain conditions.

The main limit in our research is that there is no active control group for EA (e.g., non-acupoint EA stimulation or acupuncture needle insertions without electrical stimulation). As procedures involving any kind of skin stimulation have been considered not valid as insert controls to acupuncture because they evoke activity in cutaneous afferent nerves, resulting in alleviation of the affective component of pain. Particularly, the present study does not focus on the specificity of the analgesic effect of the acupoint. In fact, the other two groups (CON group and CCI group) underwent the same constraining procedure. As neither the ipsilateral nor the contralateral PWLs were influenced by the repeated constraining process in CCI rats, this may allow us to reduce the number of control animals used in future studies.

## Conclusion

Our data showed that four successive sessions of daily EA treatment induced a time-dependent cumulative analgesic effect, which persists for at least 3 days in a rat model of neuropathic pain. This cumulative analgesic effect may depend on the activation of the MEK1 signaling pathways in the contralateral hippocampus following peripheral nerve injury.

## Abbreviations

CCI: Chronic constriction injury
DMSO: Dimethyl sulfoxide
EA: Electroacupuncture
MEK: Mitogen-activated protein kinase kinase
PWL: Paw withdrawal latency
